# Child, maternal, and adult mortality in Sierra Leone: nationally representative mortality survey 2018–20

**DOI:** 10.1016/S2214-109X(21)00459-9

**Published:** 2021-11-25

**Authors:** Ronald Carshon-Marsh, Ashley Aimone, Rashid Ansumana, Ibrahim Bob Swaray, Anteneh Assalif, Alimatu Musa, Catherine Meh, Francis Smart, Sze Hang Fu, Leslie Newcombe, Rajeev Kamadod, Nandita Saikia, Hellen Gelband, Amara Jambai, Prabhat Jha

**Affiliations:** a Ministry of Health and Sanitation, Government of Sierra Leone, Freetown, Sierra Leone; b Centre for Global Health Research, Unity Health Toronto and Dalla Lana School of Public Health, University of Toronto, Toronto, ON, Canada; c Njala University, Bo, Sierra Leone; d International Institute of Population Sciences, Mumbai, India

## Abstract

**Background:**

Sierra Leone's child and maternal mortality rates are among the highest in the world. However, little is known about the causes of premature mortality in the country. To rectify this, the Ministry of Health and Sanitation of Sierra Leone launched the Sierra Leone Sample Registration System (SL-SRS) of births and deaths. Here, we report cause-specific mortality from the first SL-SRS round, representing deaths from 2018 to 2020.

**Methods:**

The Countrywide Mortality Surveillance for Action platform established the SL-SRS, which involved conducting electronic verbal autopsies in 678 randomly selected villages and urban blocks throughout the country. 61 surveyors, in teams of four or five, enrolled people and ascertained deaths of individuals younger than 70 years in 2019–20, capturing verbal autopsies on deaths from 2018 to 2020. Centrally, two trained physicians independently assigned causes of death according to the International Classification of Diseases (tenth edition). SL-SRS death proportions were applied to 5-year mortality averages from the UN World Population Prospects (2019) to derive cause-specific death totals and risks of death nationally and in four Sierra Leone regions, with comparisons made with the Western region where Freetown, the capital, is located. We compared SL-SRS results with the cause-specific mortality estimates for Sierra Leone in the 2019 WHO Global Health Estimates.

**Findings:**

Between Sept 1, 2019, and Dec 15, 2020, we enrolled 343 000 people and ascertained 8374 deaths of individuals younger than 70 years. Malaria was the leading cause of death in children and adults, nationally and in each region, representing 22% of deaths under age 70 years in 2020. Other infectious diseases accounted for an additional 16% of deaths. Overall maternal mortality ratio was 510 deaths per 100 000 livebirths (95% CI 483–538), and neonatal mortality rate was 31·1 deaths per 1000 livebirths (95% CI 30·4–31·8), both among the highest rates in the world. Haemorrhage was the major cause of maternal mortality and birth asphyxia or trauma was the major cause of neonatal mortality. Excess deaths were not detected in the months of 2020 corresponding to the peak of the COVID-19 pandemic. Half of the deaths occurred in rural areas and at home. If the Northern, Eastern, and Southern regions of Sierra Leone had the lower death rates observed in the Western region, about 20 000 deaths (just over a quarter of national total deaths in people younger than 70 years) would have been avoided. WHO model-based data vastly underestimated malaria deaths and some specific causes of injury deaths, and substantially overestimated maternal mortality.

**Interpretation:**

Over 60% of individuals in Sierra Leone die prematurely, before age 70 years, most from preventable or treatable causes. Nationally representative mortality surveys such as the SL-SRS are of high value in providing reliable cause-of-death information to set public health priorities and target interventions in low-income countries.

**Funding:**

Bill & Melinda Gates Foundation, Canadian Institutes of Health Research, Queen Elizabeth Scholarship Program.

## Introduction

In 2019, the UN estimated life expectancy at birth in Sierra Leone to be 54 years,^1^ which ranked close to the lowest of any country. Progress in reducing premature mortality in Sierra Leone and other low-income and middle-income countries (LMICs) depends on continuous and reliable measurement of the major causes of death.^2^, ^3^ However, since many deaths in such countries continue to occur not in facilities but at home and without medical attention at the time of death, information on the major causes of death is lacking. In Sierra Leone, only 25% of all deaths are reported through the centralised vital statistics system, and no comprehensive cause-of-death information is available.^4^ The 2015 census,^5^ various demographic surveys, and model-based estimates suggest that Sierra Leone's under-5 child mortality rate (122 per 1000 livebirths) and maternal mortality ratio (1120 per 100 000 livebirths) are among the highest in the world.^6^, ^7^, ^8^

To fill this knowledge gap, the Ministry of Health and Sanitation of Sierra Leone (MOHS) launched the Sierra Leone Sample Registration System (SL-SRS) of births and deaths, with cause of death assigned with electronic verbal autopsies (e-VAs), as part of the Countrywide Mortality Surveillance for Action, a national platform to generate and disseminate mortality data that are directly relevant to national health priorities. A complete diagnostic autopsy provides conclusive evidence on cause of death for deaths occurring in hospitals, but their use is limited by insufficient diagnostic and laboratory capacity, as well as cultural and religious norms.^9^ In LMICs, verbal autopsies are acceptable alternatives to provide cause of death information.^2^, ^10^

**Research in context**

**Evidence before this study**

We searched PubMed and Google Scholar using the search terms “mortality”, “causes of death”, and “Sierra Leone” for articles published up to May 31, 2021, without language and date restrictions. We noted over 300 publications, including 144 about Ebola. No nationally representative mortality studies that included cause-of-death information were found, although overall child and maternal mortality rates were reported in the 2015 Census of Sierra Leone and in demographic surveys.

**Added value of this study**

The Sierra Leone Sample Registration System marks the establishment of an ongoing and sustainable demographic survey for births and causes of death in Sierra Leone, which has among the lowest life expectancies in the world. This first report on mortality represents a baseline of far more reliable evidence on the causes and circumstances of death than has existed before. The distribution of causes observed in this first report was substantially different (especially for adult malaria deaths) from other current estimates drawn from models or limited surveys.

**Implications of all the available evidence**

This baseline report on causes of death in Sierra Leone in 2020 gives the government and its international partners a firm basis for setting disease control priorities to improve the health of children, mothers, and other adults, and for reducing the large burden of premature deaths.

The Sierra Leone patterns of premature mortality (defined as deaths before age 70 years,^11^ following the global life expectancy in 2010 and the expected lifespan of 73·4 years in 2019),^12^ as well as the design and implementation of nationwide cause-of-death studies, are relevant to many similar settings in Africa and Asia.^2^, ^13^ In this study, we report cause-specific mortality from the first SL-SRS round, done during 2019–20, representing deaths from 2018 to 2020.

## Methods

### SL-SRS sampling frame

The SL-SRS covers about 5% of the country's total population of about 7·3 million, substantially more than any previous mortality survey in the country. The SL-SRS was designed to generate separate urban and rural district-level estimates of annual age-specific and cause-specific mortality rates every 3 years (appendix p 2). The MOHS is the lead implementer, supported by Njala University (Bo, Sierra Leone) and the Centre for Global Health Research at the University of Toronto (Toronto, ON, Canada).

**Figure 1:**
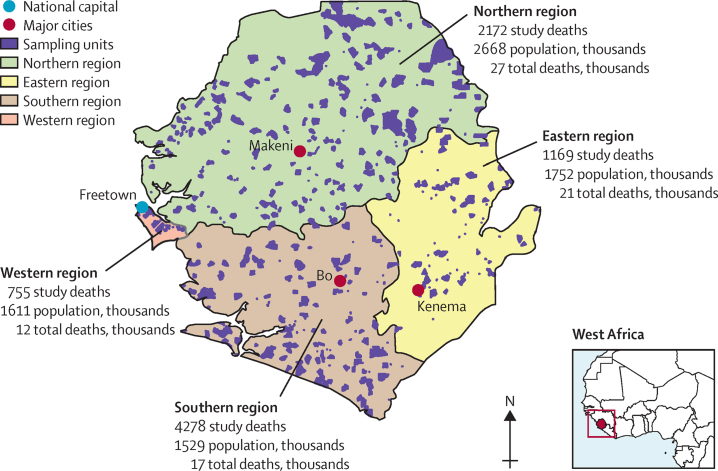
Map of Sierra Leone showing enumeration areas, regions, and numbers of study deaths Map of Sierra Leone shows the four regions and main cities of the country with the corresponding study deaths, estimated total deaths under age 70 years (in thousands), and individuals younger than 70 years (in thousands) by region.

At the start of the study, Sierra Leone had four regions (Western, Northern, Southern, and Eastern) and 16 districts (figure 1). The sample size calculation accounted for the population age structure, household size, probable response rates (96%), and an expectation of about 1% of the population dying each year.^1^ By use of a relative SE target of 0·05, the sample size was estimated to be about 45 000 households. We increased this to 60 000 households distributed among the 16 districts, for an effective relative SE of 0·043 (appendix p 3). District-level household sample sizes were calculated on the basis of the 2015 Sierra Leone census^5^ for the then 14 districts, plus two new districts created since then. This study does not cover the Northwest region, newly carved from the previous Northern and Western regions, but future reports will include all five regions.

The main sampling units are census enumeration areas, representing urban census blocks or villages, with urban and rural enumeration areas chosen in proportion to the urban and rural populations in each district. Each district's available urban and rural sampling units were divided into low, medium, and high strata according to a development index based on the 2015 census data^5^ that recorded the presence of clean fuel, clean water, concrete roofs, and improved toilets, and the percentage of literate women. We included an equal number of households from each development stratum. Enumeration areas were randomly selected in each district until the target household sample size was attained, for a total of 678 geographically dispersed enumeration areas covering an estimated population of about 340 000. The MOHS Ethics and Scientific Review Committee granted ethics approval and written informed consent was obtained from participants.

### Enumeration, baseline survey, and e-VA

After completing a pilot project in Bo District—the Bo Healthy City Study—national scale-up of the SL-SRS began on Sept 1, 2019, and the enumeration, baseline survey (reference data), and e-VA of deaths that occurred from 2018 to 2020 were completed by Dec 15, 2020. The SL-SRS instrument has three modules—enumeration, verbal autopsy, and resampling—which were thoroughly tested during the pilot phase in Bo. The e-VA uses the 2016 WHO Verbal Autopsy Standard tool, version 1.5.1,^14^ for neonates, children, adults, and stillbirths. The tool runs on laptops with an offline application that has an embedded database, eliminating the need for an internet connection. Data are validated as they are entered by the e-VA. The e-VA application has built-in GPS tracking and audio recordings and does random resampling in about 10% of households. Resampling entails reviewing verbal autopsy records for quality control and consistency.

Multiple teams of four or five full-time trained surveyors were assigned to each region, with 61 surveyors in total. Teams would typically complete enumeration, e-VA, and resampling for an enumeration area in about a week and then move to a new enumeration area. Before starting work in any area, a MOHS district social mobilisation team led sensitisation of local village leaders and community members. The survey had two stages: first, surveyors went house to house enumerating the population and identifying deaths at all ages during the preceding 3 years. Surveyors then returned to households reporting deaths and did e-VAs. We restricted e-VAs to deaths under age 70 years because of higher levels of misclassification of the causes of death at older ages compared with those in childhood and middle age.^2^, ^13^ In Sierra Leone, most deaths occur before age 70 years,^1^ representing premature mortality.^11^ Resampling occurred after the e-VAs were completed in each enumeration area through a central and anonymous reassignment of the sampled households to other team members for reinterview by use of randomly selected questions from the e-VA. Additional quality control involved central weekly review of each field staff's e-VAs, which were audiotaped.

### Central cause of death determination and collation

We assigned each death an International Classification of Diseases (tenth edition, ICD-10) code referring to the underlying cause of death on the basis of review of the e-VA and narrative independently by two of 11 physicians specially trained on death certification and ICD coding of verbal autopsy. These physicians assigned causes independently and anonymously, with reconciliation of differences and masked adjudication by a senior physician, identical to the procedure used in the Indian Million Death Study.^15^, ^16^ Maternal deaths underwent a second central panel of obstetrical review to arrive at the final diagnoses. Central coding was electronic (online or offline, with connectivity needed only once every few days to synchronise data), enabling assignment of causes within days of data collection.

We grouped coded deaths according to a standard death classification system based on the WHO Global Health Estimates^7^ and adapted from the Indian National Burden Estimates.^15^ This system comprises 45 distinct groups of causes drawn from the entire range of 474 unique ICD-10 codes captured in the study. We tabulated direct (underlying) and indirect (contributory) causes of maternal deaths following WHO–ICD maternal death coding guidelines.^17^ We derived sex-specific national absolute death totals for children (aged 0–28 days for neonates, 1 month to 4 years, and 5–14 years) and adults (aged 15–29 years and 30–69 years) using the two 5-year averages (2015–19 and 2020–24) of total deaths within each age group from the UN World Population Prospects (2019).^1^ Similarly, we estimated livebirths (for ages younger than 5 years) from the same source. We calculated neonatal death estimates by applying the proportional neonatal mortality rate published by UNICEF for 2019.^18^ Stillbirths have been presented separately and the national stillbirth rate is expressed in terms of total births (livebirths plus stillbirths). Deaths in Bo District were over-represented in absolute numbers from inclusion of the Bo Healthy City Study results; however, we applied sample weights to correctly represent this district and ensure that overall national and regional totals were not distorted.

### Statistical analysis

We applied the SL-SRS sample-weighted proportions of deaths to the UN Population Division death totals, and partitioned data for the four Sierra Leone regions on the basis of the SL-SRS proportions of deaths and those from the 2015 Census^5^ and 2019 Demographic and Health Survey (DHS).^6^ We resummed the four regional totals to the national UN estimates to ensure consistency. The period risk for each major cause of death was the proportion of the UN life-table-based, age-specific mortality rate.^1^ We based 95% CIs for the age-specific and cause-specific rates on the Δ method.^19^ The calculation of avoidable mortality for each major age group compared the urban rates of the Western region (which is predominantly urban) with the urban rates in the other regions. We assessed monthly mortality levels in 2020 compared with those of preceding years for excess deaths due to COVID-19. To investigate malaria, we stratified malaria deaths into 4-year age spans from ages 15–60 years, 60–69 years, and separately for under-5-year-olds. Finally, we also compared SL-SRS results with the cause-specific mortality estimates for Sierra Leone in the 2019 WHO Global Health Estimates.^7^ These WHO estimates combine model-based data with WHO disease-specific programme information. Analyses were done in Stata, version 16 (summary statistics and ICD-10 codes), and R, version 4.05 (spatial analyses of geocoded units).

### Role of the funding source

The funders of the study had no role in study design, data collection, data analysis, data interpretation, or writing of the report.

## Results

Between Sept 1, 2019, and Dec 15, 2020, 678 enumeration areas were included in the study, with an enumerated population of approximately 343 000, 51% of whom lived in rural areas (figure 1; appendix p 4). After exclusion for missing records, ICD-10 codes were assigned to 8374 deaths by two independent physicians, with 1558 (19%) deaths undergoing reconciliation alone and 1978 (24%) undergoing reconciliation and adjudication. With some variation across age groups, 4702 (56%) deaths occurred in rural areas and 3965 (47%) of all deaths occurred at home. Results are presented by age group and separately for maternal deaths. Details of study deaths and national and regional totals are presented in the appendix (pp 5–10).

Of 415 neonatal deaths among the enumerated households, 229 (55%) were male and 186 (45%) were female, 122 (29%) occurred at home, and 241 (58%) occurred in rural settings. The UN-estimated neonatal death rate of 31·1 deaths per 1000 livebirths (95% CI 30·4–31·8) yielded 8018 neonatal deaths in 2020 nationwide. Of these, over 80% (6564) arose from birth asphyxia or trauma (combined, representing birth injury), sepsis and other infections, and prematurity or low birthweight, with similar results in each region. We also identified 154 stillbirths: 75 (49%) male and 79 (51%) female, 20 (13%) occurring at home, and 99 (64%) occurring in rural settings. Applying the weighted proportion of these deaths to the denominator of livebirths and stillbirths yielded 4104 stillbirths nationally.

For children younger than 5 years (minus neonatal deaths), 19 700 deaths occurred nationally (76·3 deaths per 1000 livebirths, 95% CI 75·3–77·4). At these ages, the leading causes among 2322 study deaths in the enumerated households were malaria and other infectious diseases, accounting for more than 60% of the total (table 1), or about 13 000 deaths from these conditions per year nationally. The overall risk of death for children dying before age 5 years was high, with 3·1% dying during the first month of life and 7·6% at ages 1–59 months (table 1).

**Table 1:** Causes of deaths among neonates (aged 0–28 days), children (1–59 months), and maternal causes (15–49 years) in Sierra Leone, 2018–20

			**Study deaths (male/female)**	National annual deaths*	**Percentage of national totals**†	**Risk of death, %**	**Annual mortality rate, per 1000 livebirths**‡	**Lifetime death risk, %**	**Maternal mortality ratio, per 100 000 livebirths**‡
Stillbirths			154 (75/79)	4104	34%	NA	15·6 (15·1–16·1)	..	..
Neonatal deaths			..	..	..	..	..	..	..
	Birth asphyxia and birth trauma		129 (77/52)	2550	32%	1·0%	9·9 (9·5–10·3)	..	..
	Sepsis and other infections		118 (65/53)	2418	30%	0·9%	9·4 (9·0–9·8)	..	..
	Prematurity and low birthweight		89 (42/47)	1596	20%	0·6%	6·2 (5·9–6·5)	..	..
	Other non-communicable causes§		27 (17/10)	531	7%	0·2%	2·1 (1·9–2·2)	..	..
	Pneumonia		16 (7/9)	267	3%	0·1%	1·0 (0·9–1·2)	..	..
	Ill-defined or cause unknown		36 (21/15)	656	8%	0·3%	2·5 (2·4–2·7)	..	..
	All neonatal deaths		415 (229/186)	8018	100%	3·1%	31·1 (30·4–31·8)	..	..
Deaths at 1–59 months			..	..	..	..	..	..	..
	Malaria		855 (429/426)	7417	38%	2·9%	28·7 (28·1–29·4)	..	..
	Other infectious and parasitic causes¶		625 (309/316)	5545	28%	2·1%	21·5 (20·9–22·1)	..	..
	Pneumonia		209 (101/108)	1481	7%	0·6%	5·7 (5·5–6·0)	..	..
	Diarrhoea		182 (93/89)	1606	8%	0·6%	6·2 (5·9–6·5)	..	..
	Other non-communicable causes		84 (36/48)	697	4%	0·3%	2·7 (2·5–2·9)	..	..
	Injuries		82 (45/37)	697	4%	0·3%	2·7 (2·5–2·9)	..	..
	Meningitis or encephalitis		55 (30/25)	319	2%	0·1%	1·2 (1·1–1·4)	..	..
	Measles		31 (16/15)	243	1%	0·1%	0·9 (0·8–1·1)	..	..
	Acute bacterial sepsis and severe infections		18 (9/9)	145	1%	0·1%	0·6 (0·5–0·7)	..	..
	HIV or AIDS		18 (10/8)	168	1%	0·1%	0·7 (0·6–0·8)	..	..
	Nutritional diseases		14 (7/7)	108	1%	0·0%	0·4 (0·3–0·5)	..	..
	Congenital anomalies		10 (3/7)	69	<1%	0·0%	0·3 (0·2–0·3)	..	..
	Sickle-cell disorders		9 (7/2)	82	<1%	0·0%	0·3 (0·3–0·4)	..	..
	Fever of unknown origin		46 (22/24)	427	2%	0·2%	1·7 (1·5–1·8)	..	..
	Ill-defined or cause unknown		84 (43/41)	699	3%	0·3%	2·7 (2·5–2·9)	..	..
	All 1–59 months deaths		2322 (1160/1162)	19 704	100%	7·6%	76·3 (75·3–77·4)	..	..
Maternal deaths			..	..	..	..	..	..	..
	Direct causes		112	1046	78%	..	..	2·7%	405 (381–430)
		All haemorrhage	36	336	25%	..	..	0·9%	130 (117–145)
		Infection and sepsis	22	205	15%	..	..	0·5%	80 (70–92)
		Hypertensive disorders	15	140	9%	..	..	0·4%	54 (46–64)
		Abortion	13	121	7%	..	..	0.3%	47 (39–56)
		Other specified causes	10	93	7%	..	..	0·2%	36 (29–44)
		Ill-defined	16	149	14%	..	..	0·4%	58 (49–68)
	Indirect causes		29	271	22%	..	..	0·7%	105 (93–118)
	All maternal deaths		141	1317	100%	..	..	3·4%	510 (483–538)

Data are n, %, or rate (95% CI). NA=not applicable.

* The national total for stillbirths was calculated as the proportion of stillbirths (from this study) out of total deaths in the perinatal period in 2019 applied to the 2020 population; national total for maternal deaths was estimated using the UN 2020 average for deaths among women aged 15–49 years.

† Percentages are weighted for district and for urban or rural residence; for stillbirths the percentage is of the 12 122 combined total of neonatal and stillbirth deaths nationally; for maternal deaths, the percentage is of all maternal deaths of women aged 15–49 years.

‡ Livebirths for 2020 were estimated by use of the 10-year average (2015–25) from the UN World Population Prospects (2019); stillbirth rate per 1000 includes livebirths and stillbirths in the denominator.

§ Other non-communicable causes include congenital anomalies (28%), neonatal jaundice (20%), and feeding problems (20%).

¶ Other infectious and parasitic causes were mostly other and unspecified infectious diseases (93%).

We recorded 141 maternal deaths in 2018–20 (table 1), accounting for nearly 9% of all deaths among women aged 15–49 years and an estimated 1317 maternal deaths nationwide. The major causes were haemorrhage, post-partum sepsis, and hypertensive disorders (63% of all direct causes of maternal deaths). Of the major indirect causes, the highest proportion of deaths was due to communicable diseases (20 [14%]). The overall maternal mortality rate was 510 deaths per 100 000 livebirths per year (95% CI 483–538), representing a lifetime risk of maternal death of 3·4% (table 1).

Death rates were substantially lower among children aged 5–14 years compared with younger ages, but still, we estimate that 2·9% of children reaching age 5 years could die before age 15 years. Among the 6227 deaths occurring nationally in children aged 5–14 years, 2119 (34%) were due to malaria, followed by other infectious diseases (1464 [23%]), and non-communicable diseases (643 [10%]). Injuries accounted for about 10% of the national deaths in this age group (table 2).

**Table 2:** Causes of death among children (5–14 years) and adults (15–29 years and 30–69 years) in Sierra Leone, 2018–20

	**Study deaths (male/female)**	National annual deaths*	**Percentage of national totals**†	**Mortality rate, per 100 000 population**‡	**Period risk, %**
**5–14 years**					
Malaria	250 (140/110)	2119	34%	99·1 (95·0–103·5)	0·98%
Other infectious and parasitic causes§	166 (81/85)	1464	23%	68·5 (65·1–72·1)	0·67%
Other non-communicable causes§	86 (52/34)	643	10%	30·1 (27·9–32·5)	0·29%
Injuries	88 (60/28)	652	10%	30·5 (28·3–32·9)	0·29%
Diarrhoea	60 (41/19)	496	8%	23·2 (21·3–25·3)	0·23%
Pneumonia	32 (16/16)	221	4%	10·3 (9·1–11·8)	0·12%
Sickle-cell disorders	29 (14/15)	285	5%	13·3 (11·9–15·0)	0·14%
Meningitis or encephalitis	17 (9/8)	128	2%	6·0 (5·0–7·1)	0·06%
Acute bacterial sepsis and severe infections	10 (5/5)	95	2%	4·5 (3·6–5·4)	0·06%
Ill-defined or cause unknown	16 (7/9)	124	2%	5·8 (4·9–6·9)	0·06%
All 5–14 years deaths	754 (425/329)	6227	100%	291·3 (284·3–298·7)	2·90%
**15–29 years**					
Malaria	239 (129/110)	2483	20%	106·0 (101·9–110·2)	1·57%
Other infectious conditions	141 (70/71)	1311	11%	56·0 (53·0–59·1)	0·87%
Injuries¶	104 (83/21)	1181	10%	48·3 (47·7–53·4)	0·79%
Road traffic accidents	88 (59/29)	797	6%	34·0 (31·7–36·5)	0·47%
Diarrhoea	61 (31/30)	664	5%	28·3 (26·3–30·6)	0·39%
Acute respiratory infections	52 (27/25)	533	4%	22·7 (20·9–24·8)	0·31%
All vascular causes	47 (20/27)	473	4%	20·2 (18·5–22·1)	0·31%
HIV or AIDS and other STIs	41 (16/25)	474	4%	20·2 (18·5–22·1)	0·31%
Other digestive causes	97 (59/38)	1052	9%	44·9 (42·3–47·7)	0·71%
Severe systemic infection and other infections	64 (36/28)	579	5%	24·7 (22·8–26·8)	0·39%
Sickle-cell disorders	28 (10/18)	275	2%	11·7 (10·4–13·2)	0·16%
Cancers	26 (8/18)	306	2%	13·1 (11·7–14·6)	0·16%
Other conditions	161 (24/138)	1566	13%	66·9 (63·6–70·2)	1·02%
Ill-defined or cause unknown	42 (20/22)	560	5%	23·9 (22·0–26·0)	0·39%
All 15–29 years deaths	1192 (593/599)	12 254	100%	523·1 (513·9–532·4)	7·87%
**30–69 years**					
Malaria	547 (292/255)	4392	14%	213·8 (207·6–220·2)	7·48%
Cardiac and other vascular causes‖	375 (199/176)	3121	10%	151·9 (146·7–157·4)	5·34%
Digestive diseases‖	395 (267/128)	3295	11%	160·4 (155·0–166·0)	5·88%
Stroke	317 (171/146)	2503	8%	121·8 (117·2–126·7)	4·28%
Injuries	221 (160/61)	1920	6%	93·5 (89·4–97·7)	3·21%
Other infectious causes	203 (115/88)	2294	8%	111·6 (107·2–116·3)	4·28%
Diarrhoea	200 (101/99)	1814	6%	88·3 (84·3–92·5)	3·21%
Acute respiratory infections	173 (103/70)	1487	5%	72·4 (68·8–76·2)	2·67%
Cancers	162 (71/91)	1567	5%	76·3 (72·6–80·2)	2·67%
Road traffic accidents	146 (105/41)	1073	3%	52·2 (49·2–55·5)	1·60%
Tuberculosis	133 (91/42)	1287	4%	62·6 (59·3–66·2)	2·14%
HIV or AIDS and other STIs	96 (48/48)	886	3%	43·1 (40·4–46·1)	1·60%
Severe systemic infection and other infections	153 (74/79)	1289	4%	62·8 (59·4–66·3)	2·14%
Asthma and chronic obstructive pulmonary conditions	58 (32/26)	565	2%	27·5 (25·3–29·9)	1·07%
Nephritis and nephrosis	52 (33/19)	400	1%	19·5 (17·7–21·5)	0·53%
Sickle-cell disorders	18 (10/8)	174	1%	8·5 (7·3–9·8)	0·53%
Other medical causes	195 (65/130)	1762	6%	85·8 (81·9–89·9)	3·21%
Ill-defined and abnormal findings	92 (44/48)	907	3%	44·1 (41·4–47·1)	1·60%
All 30–69 years deaths	3536 (1981/1555)	30 736	100%	1496·2 (1479·6–1513·0)	53·45%

Data are n, %, or rate (95% CI). STI=sexually transmitted infection.

* National totals were estimated by use of the 2020 average for deaths in each age group (5–14 years, 15–29 years, and 30–69 years) from the UN World Population Prospects (2019).^1^

† Percentages are weighted for district and for urban or rural residence.

‡ Mortality rates were estimated by use of the 2020 average for population in each age group (5–14 years, 15–29 years, and 30–69 years) from the UN World Population Prospects (2019).^1^

§ Other infectious and parasitic causes include “other and unspecified infectious diseases” (63%) and tuberculosis, measles, fever of unknown origin, or HIV or AIDS (19%); other non-communicable causes include diseases of the digestive system and nutritional diseases (40%).

¶ Injuries include falls (21%), exposure to inanimate mechanical forces (12%), and contact with venomous animals and plants (19%).

‖ Cardiac and other vascular causes include ischaemic heart disease (64%) and heart failure (17%); digestive diseases include liver and alcohol-related diseases (30%), gastrooesophageal causes (24%), and diseases of the digestive system (23%).

We estimate that 7·9% of individuals reaching age 15 years could die before age 30 years, totalling 12 254 deaths per year. Malaria was the leading cause of death among young adults, accounting for 20% of deaths, or an estimated 2483 deaths nationally. Other infections, injuries, and road traffic accidents accounted for an additional 3289 (27%) deaths in this relatively healthy age group (table 2, appendix pp 5–10). Male individuals represented 56% of deaths nationally, with 39% having died at home, and 68% of deaths occurring in a rural setting.

We estimate that 53·5% of individuals reaching age 30 years could die before age 70 years (30 736 per year). Even among middle-aged and older adults, malaria caused more deaths than any other single cause, at 14% (4392) of all deaths in this age group (table 2). The next most frequent single cause was cardiac and other vascular causes (3121 [10%]). Strokes caused 2503 (8%) deaths. Cancers accounted for only 5% (1567) of all deaths. About 25% (7770) were due to other infections, including tuberculosis (1287 [4%]), severe systemic and other infections (1289 [4%]) and HIV or AIDS and other sexually transmitted infections (886 [3%]). Deaths at these ages were 56% male, 53% occurred at home, and 47% occurred in rural areas. We looked specifically for excess deaths in this age group that might indicate a higher rate of COVID-19 incidence than that reported officially in Sierra Leone. We found no observable excess from respiratory conditions during May–June, 2020, when the first COVID-19 cases were reported, notably in Freetown and the rest of the Western region^20^ compared with data from 2019 (data not shown). However, study numbers of respiratory deaths were small—only about 62 cumulatively, of which nearly two-thirds occurred in the Western region.

**Figure 2:**
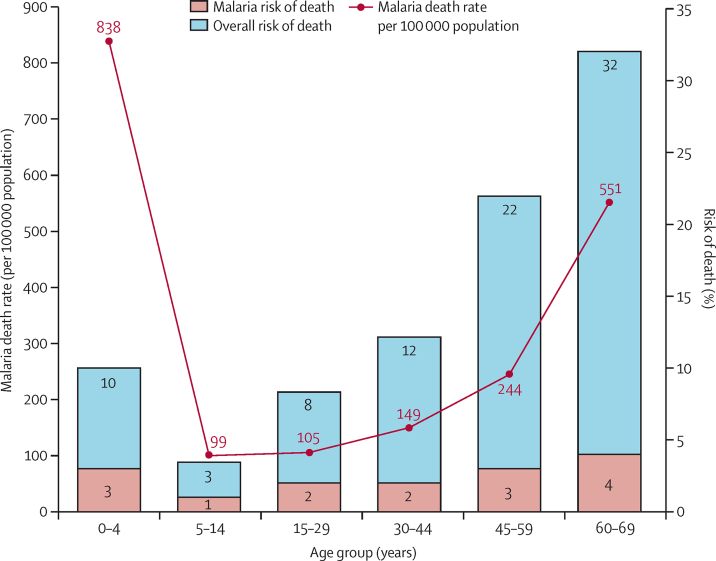
Annual malaria mortality rate by age group in Sierra Leone The overall malaria risk of death at ages 30–69 years was 7·48% and total risk of death in the same age group was 53·45%. Malaria risk of death is a proportion of the overall risk of death for each age group, with the UN life tables^1^ used to determine the overall risk of death and the Sierra Leone Sample Registration System used to determine death proportions due to malaria.

Malaria (unspecified as to being caused by *Plasmodium falciparum* or *Plasmodium vivax*) was the leading single cause of death nationally in all age groups except neonates, in both sexes, and in all four regions, although not in every age group in every region. Malaria represented 22% of deaths under age 70 years in 2020. Other infectious diseases (which could be misclassified as malaria or vice versa)^16^ accounted for an additional 16% of deaths. Even in the largely urban Western region, malaria was a leading cause of death (appendix p 9). The age-specific patterns of malaria deaths showed a roughly U-shaped curve (figure 2), with the expected high mortality among young children and low rates in older children and younger adults, but rising in later middle and older age groups. For example, at ages 45–59 years, the overall risk of death in both sexes was 22·1% and the risk of death from malaria was 2·9%, or one in seven deaths in this age range.

**Figure 3:**
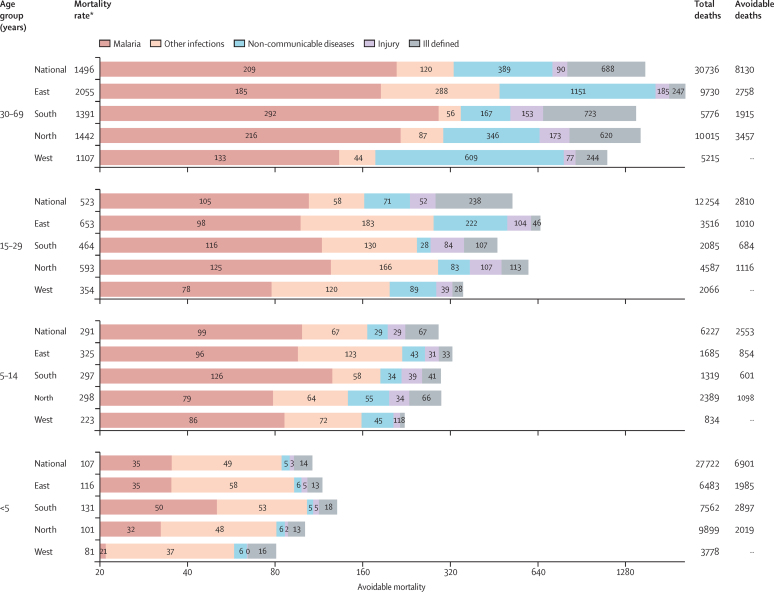
Avoidable mortality estimates for three regions compared to the Western region for children younger than 5 years and for ages 5–69 years *For children younger than 5 years, estimates are per 1000 livebirths. For age group 5–14 years, 15–29 years, and 30–69 years, estimates are per 100 000 population.

The overall levels of childhood mortality under age 5 years were variable across regions, with a marked excess of malaria deaths, particularly in the Southern region (figure 3). These analyses suggest that, if the whole of Sierra Leone had the child death rates of the Western region, about 6900 of the 27 700 deaths under age 5 years would have been avoided. Striking differences in the proportion of avoidable deaths were also seen at older ages. The Eastern region had notably high adult death rates, driven by infections other than malaria and non-communicable diseases. Overall, the proportions of avoidable deaths for Sierra Leone were about 27% (about 13 000 of 49 000 deaths at ages 5 years or older), with about 41% of deaths at ages 5–14 years being avoidable. The summation of the national totals of avoidable deaths for the four age groups yielded an estimate of slightly over 20 000 avoidable deaths, or just over a quarter of total deaths before age 70 years (figure 3).

We compared SL-SRS data with WHO estimates of cause-specific mortality (table 3). We found a nearly two-times higher total for malaria deaths within the SL-SRS compared with the WHO estimate (16 718 from SL-SRS *vs* 9111 from WHO). The maternal mortality rate per 100 000 livebirths was lower than the estimate from the 2019 DHS, which reported 717,^6^ and substantially lower than the 2017 WHO estimate of 1120.^8^ SL-SRS totals for non-communicable diseases showed fewer deaths from cancer and congenital anomalies than the WHO estimates. Injury deaths overall were similar between SL-SRS and WHO, but SL-SRS had more deaths from falls and fewer from road traffic accidents, suicide, and interpersonal violence (table 3). Importantly, WHO estimates contained no measure of ill-defined or unclassifiable deaths, but it is unlikely that there would not be some ill-defined deaths in a real-world setting,^2^ whereas SL-SRS found this category to include 1825 (2·4%) deaths of people younger than 70 years.

**Table 3:** Comparison of COMSA and WHO cause-of-death proportions and counts in Sierra Leone, 2018–20

		**COMSA Sierra Leone national deaths**	**WHO estimated total deaths**
All causes		76 939 (100%)	76 939 (100%)
Communicable, maternal, perinatal, and nutritional causes		51 784 (67·3%)	48 060 (62·5%)
	Tuberculosis	1691 (2·2%)	3062 (4·0%)
	STIs (including HIV and AIDS)	1482 (1·9%)	3678 (4·8%)
	Diarrhoea	4440 (5·8%)	6111 (7·9%)
	Malaria	16 718 (21·7%)	9111 (11·8%)
	Respiratory infections	4386 (5·7%)	8244 (10·7%)
	Other infectious and parasitic diseases	11 921 (15·5%)	5051 (6·6%)
Non-communicable causes		16 909 (22·0%)	21 172 (27·5%)
	All cancer	1827 (2·4%)	3311 (4·3%)
	Sickle-cell anaemia	781 (1·0%)	909 (1·2%)
	Stroke	2876 (3·7%)	3044 (4·0%)
	Ischaemic heart disease	3482 (4·5%)	2864 (3·7%)
	Liver and alcohol-related disease	1361 (1·8%)	1453 (1·9%)
	Congenital anomalies	242 (0·3%)	2076 (2·7%)
Injuries		6421 (8·3%)	7707 (10·0%)
	Road traffic accidents	2061 (2·7%)	3230 (4·2%)
	Falls	1032 (1·3%)	285 (0·4%)
	Drowning	432 (0·6%)	480 (0·6%)
	Suicide	44 (0·1%)	618 (0·8%)
	Interpersonal violence	211 (0·3%)	832 (1·1%)
Ill-defined or cause unknown		1825 (2·4%)	..

Data are n (%), with the percentages weighted by the sampling probability. WHO total deaths were 56 775 compared with 76 939 in the UN Population Division for ages 0–69 years. WHO totals^7^ are scaled to UN Population Division totals.^1^ WHO has produced two incompatible estimates of total maternal deaths in 2020: 1192 (from the Global Health Estimates)^7^ and 2811 (from the multi-agency estimates).^8^ COMSA=Countrywide Mortality Surveillance for Action. STI=sexually transmitted infection.

## Discussion

About 63% of people in Sierra Leone die prematurely, before age 70 years, mostly from preventable or treatable causes. This single fact illuminates the high value of reliable cause-of-death information for improving life expectancy in Sierra Leone and in other low-income countries in Africa and Asia.^2^, ^13^

The SL-SRS represents the most complete and nationally representative accounting of causes of death to date in Sierra Leone. It is the first report from a sampling system that will continue to monitor deaths and their causes over the long term, with an increasing proportion of medically certified deaths for those that occur in facilities. Previous and other current sources of cause-specific mortality, including the annual reports from UN agencies, have had to rely on information from deaths in health facilities, which are often not medically certified, and which are seldom representative, used along with modelling and data from what are considered similar countries to produce estimates. The SL-SRS should improve public health planning and results tracking.^21^ Parallel efforts by the Child Health and Mortality Prevention Surveillance (CHAMPS) study will provide information on histological and microbiological testing of deceased children to establish detailed and more specific causes (such as subtypes of pneumonia and diarrhoeal deaths), as well as comorbidities, and to assess the contribution of key conditions (notably malnutrition) by use of minimally invasive tissue sampling.^22^ CHAMPS plans to survey about 300 deceased children in Sierra Leone by 2022.^23^

The most striking finding of SL-SRS is that 22% of all deaths in 2020 were assigned to malaria, and that malaria was the leading cause of death in all age groups except neonates. These findings are consistent with analyses from the past decade, but which are still controversial, that challenge the long-held view that few malaria deaths occur after childhood in highly endemic areas.^16^, ^24^ The high malaria death toll among adults accounted for most of the large discrepancy between SL-SRS and WHO estimates. Moreover, WHO estimates do not suggest missing deaths from other acute infections, which has been suggested by commentators in response to previous reports of high levels of adult malaria mortality.^25^ Misclassification, under-reporting, or over-reporting from studies and models that inform WHO estimates might explain these differences. Further exploration of acute febrile deaths in adults with biological testing^19^ is warranted.

Sierra Leone is reported to have one of the highest maternal death rates in the world.^8^ We found about 1300 maternal deaths, corresponding to a rate of about 510 per 100 000 livebirths—still high, but only 40% of the WHO estimate. Sierra Leone's facility-based maternal death reporting system documented an annual average of 574 maternal deaths in 2017–19, and the SL-SRS documented that nearly three-quarters of maternal deaths occurred in facilities or on the way to facilities. Collectively, these data warrant a substantial downward revision of WHO estimates of the country's maternal death totals.

The most important implications of these SL-SRS findings are for aligning the mortality burden with health programmes and expenditures, which might entail reconsidering resource allocation for ongoing efforts and new programmes. The national maternal and child health programmes need to address stillbirths, which might well represent avoidable deaths just before delivery.^26^ Similarly, the relative contribution of malaria and other acute febrile infections suggests that additional efforts to scale up prompt diagnosis and treatment of acute fevers for adults and children are needed. The leading causes of obstetric deaths arose from haemorrhage or related conditions that are amenable to emergency obstetric services, including safe delivery within facilities and access to safe blood transfusion. Finally, while much attention of existing programmes is on maternal and child health, SL-SRS identified the very high levels of avoidable deaths in middle age, notably from malaria, that should form the basis for greater investments in adult health. Regular reporting of SL-SRS data over the coming years should clarify the rate of change and hopefully encourage continued efforts at improvement, as most of these premature deaths are preventable with modest investments.^27^ The absence of excess deaths during the months corresponding to the 2020 COVID-19 peak in cases is consistent with reported cumulative seroprevalence lower than 3% among adults (before the June–July 2021 viral wave).^28^ Importantly, the SL-SRS provides a system to track increases in mortality that might arise from subsequent viral waves of COVID-19.

The strengths of our study are its nationally representative and distributed sampling (nearly 680 small sampling areas) and the use of a robust and well validated e-VA instrument and dual independent physician coding (to be complemented by addition of InSilicoVA and InterVA algorithm-based diagnoses),^10^ as well as more detailed analyses of major conditions, such as the risks during intrapartum, interpartum, and neonatal periods for neonatal deaths.^29^ Nonetheless, our study has some limitations. First, even with substantial advances in methods and technology that we applied to SL-SRS, e-VAs have limitations in assigning cause of death. Some conditions, such as respiratory conditions, febrile illnesses, and some cancer sites, are particularly hard to diagnose on e-VA.^10^ Second, the SL-SRS takes in about 5% of the population, which might lead to a wide range of uncertainty, especially for less common causes. Although e-VAs yield only crude information on causes of death, the data produced by the SL-SRS is an order of magnitude better than previous estimates available for the country.^6^, ^7^, ^8^ The findings on the burden of premature deaths and causes should be useful indicators for countries with profiles similar to Sierra Leone, but they cannot substitute for data collected directly for a given country.

In conclusion, SL-SRS will provide direct evidence on causes of death and mortality estimates to the MOHS and other key stakeholders in Sierra Leone, decreasing reliance on more uncertain model-based estimates. At the same time, direct estimates should help to improve model-based estimates for Africa, which has an unacceptably low ratio of actual versus modelled data.^2^

## Data sharing

The grouped level Countrywide Mortality Surveillance for Action mortality data are available online (www.comsasl.org) and the full dataset is available upon application on the website.

## Declaration of interests

We declare no competing interests.
