# Supplementary material for: Child, maternal, and adult mortality in Sierra Leone: nationally representative mortality survey 2018–20
Source: Lancet Glob Health. 2021 Nov 25;10(1):e114–23. doi: 10.1016/S2214-109X(21)00459-9 (PMC8672062; doi:10.1016/S2214-109X(21)00459-9)
Supplement: Supplementary appendix [file mmc1.pdf]

THE LANCET
Global Health

Supplementary appendix

This appendix formed part of the original submission and has been peer reviewed. We post it as supplied by the authors.

Supplement to: Carshon-Marsh R, Aimone A, Ansumana R, et al. Child, maternal, and adult mortality in Sierra Leone: nationally representative mortality survey 2018–20. *Lancet Glob Health* 2021; published online Nov 25. http://dx.doi.org/10.1016/S2214-109X(21)00459-9.

Appendix to:

Child, maternal and adult mortality in Sierra Leone: Nationally representative mortality survey 2018-2020

Ronald Carshon-Marsh*, Ashley Aimone, Rashid Ansumana*, Ibrahim Bob Swaray, Anteneh Assalif, Alimatu Musa, Catherine Meh, Francis Smart, Sze Hang Fu, Leslie Newcombe, Rajeev Kamadod, Nandita Saikia, Hellen Gelband, Amara Jambai*, Prabhat Jha

Contents

Figure 1: Flowchart of SL-SRS activities……………………………………………………………………………………..2

Sample size calculation…………………………...……………………………………………………………………………….3

Table 1: Number of sampled enumeration areas and population covered by district and urban/rural residence……4

Table 2: Study deaths by age, sex, and cause, Sierra Leone……………………………………………………………5

Table 3: National Estimated deaths (2020) by age, sex and cause, Sierra Leone……………………………………6

Table 4: Eastern Region burden of disease estimates (2020) for COMSA deaths by age, sex and cause…..7

Table 5: Northern Region burden of disease estimates (2020) for COMSA deaths by age, sex and cause..8

Table 6: Western Region burden of disease estimates (2020) for COMSA deaths by age, sex and cause…9

Table 7: Southern Region burden of disease estimates (2020) for COMSA deaths by age, sex and cause.... ……10

SL-SRS FLOWCHART 2018-2020

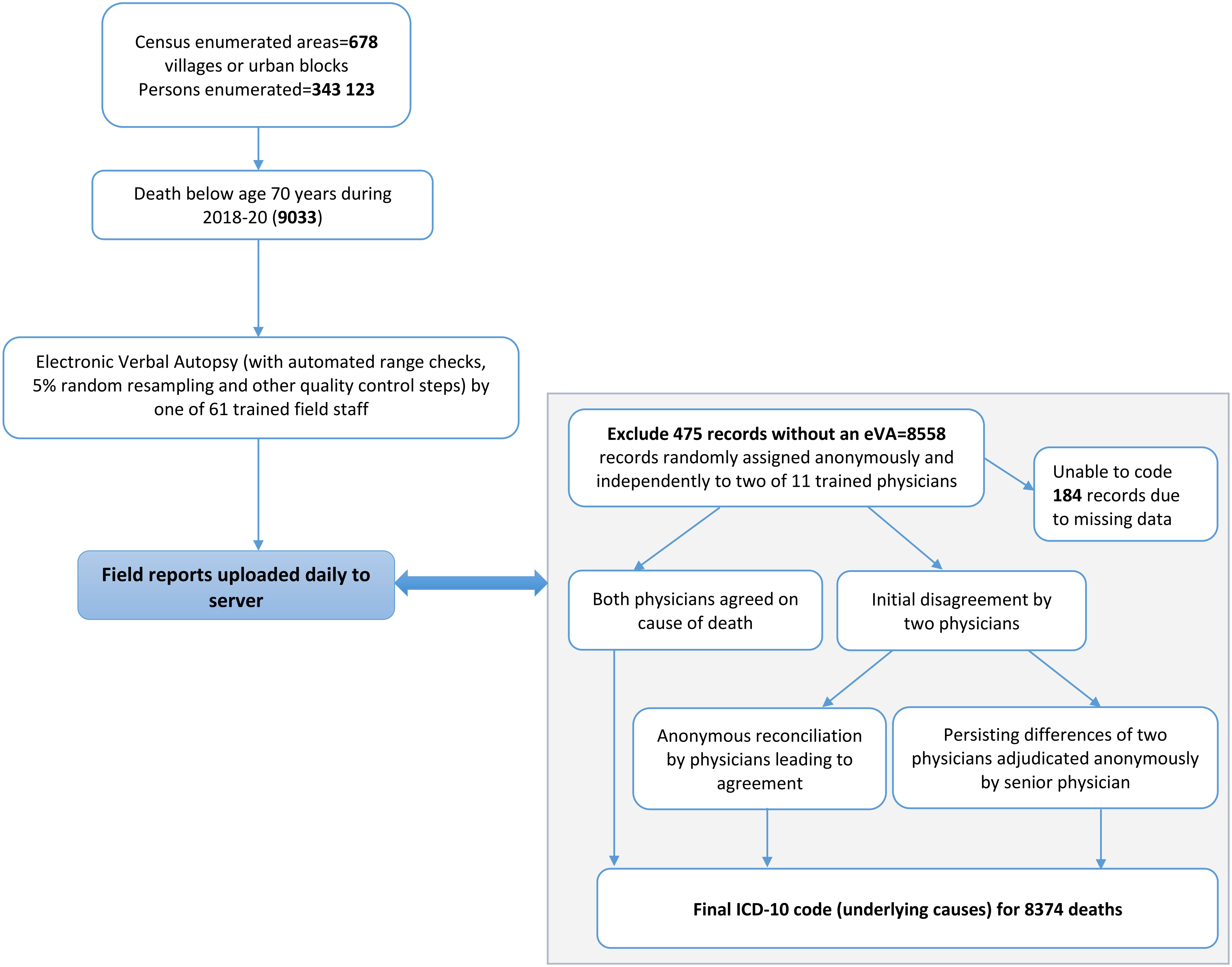

Sample size calculation

The total size of the population of Sierra Leone was 7.3 million. We assumed a household and individual response rate of 96% and proportion of death at country level was 0.01445.

The sample size was calculated from the following formula:

$$n = \text{Deft}^2 \times \frac{(1/P-1)}{\alpha^2} \Big/ (R_i \times R_h \times d)$$

Where Deft=Design effect, 1.5 by default
P=Proportion of death at country level (=0.01445)
α= Relative standard error (precision) (=0.043)
R_i=Individual standard rate, (=0.96)
R_h= Household response rate (=0.96)
K=number of eligible persons for interview (1)
The relative standard error (RSE) was calculated using the formula:

$$RSE(P) \cong SQRT\left(\frac{P(1-P)}{n}\right) / P = SQRT\left(\frac{1/P-1}{n}\right)$$

Table 1: Number of sampled enumeration areas and population covered by district and urban/rural residence

Region & District	Number of enumeration areas			Population covered			Study deaths by locality and place of death¶		
	Total	Urban	Rural	Total	Urban	Rural	Total	Rural§	Home‡
**Western Region**									
Western Area Urban	82	82	0	30,020	30,020	0	515	0	196
Western Area Rural	30	27	3	11,243	10,351	892	240	20	104
**Northern Region**									
Bombali	35	12	21	19,074	8,546	10,528	333	191	148
Karene	9	1	8	4,498	562	3,936	63	54	29
Tonkolili	48	7	41	24,689	4,026	20,663	513	434	274
Port Loko	28	8	20	9,667	3,310	6,357	112	75	69
Kambia	49	16	33	20,509	5,027	15,482	538	414	285
Koinadugu	56	17	39	23,772	7,416	16,356	326	226	163
Falaba	50	7	43	16,591	2,583	14,008	287	244	150
**Southern Region**									
Bo*	60	34	26	90,587	73,636	16,951	2,306	541	1,003
Moyamba	29	3	26	11,737	1,563	10,174	510	432	285
Pujehun	45	3	42	15,727	1,217	14,510	596	554	294
Bonthe	55	10	45	24,365	3,711	20,654	866	738	443
**Eastern Region**									
Kenema	29	14	15	14,816	8,009	6,807	558	327	239
Kailahun	27	9	18	9,087	2,567	6,520	273	202	127
Kono	46	13	33	16,741	4,426	12,315	338	250	156
**Total**	**678**	**263**	**413**	**343,123**	**166,970**	**176,153**	**8,374**	**4,702**	**3,965**

¶ Eligible deaths (<70 years of age) that underwent dual physician coding for cause of death assignment.
* The national totals of deaths and death rates are sample weighted so that Bo deaths are represented only in proportion to their contribution to national totals, not to study totals (where they are oversampled).
‡ The overall percentage of deaths occurring at home was 48%, varying by age group: neonates 29%; 1-59 month 47%; 5-14 years 39%; 15-29 years 48%; maternal deaths 15-49 years 23%; 30-69 years 53%.
§ The overall percentage of deaths occurring in rural areas was 56%, varying by age group: neonates 58%; 1-59 month 67%; 5-14 years 68%; 15-29 years 52%; maternal deaths 15-49 years 52%; 30-69 years 47%.

Table 2: Study deaths by age, sex and cause, Sierra Leone

	Sex	Both sexes	Male	Female	Male					Female				
	Age group	Total (all ages)	Total	Total	0-28 days	0-4 years	5-14 years	15-29 years	30-69 years	0-28 days	0-4 years	5-14 years	15-29 years	30-69 years
**Population, thousands (UN 2020)**		7,559	3,776	3,783		519	1,093	1,125	1,039	3,768	506	1,045	1,216	1,016
Births (UN 2020)		258,100												
Code	***Cause of death***													
	**All Causes**	**8,374**	**4,463**	**3,911**	**229**	**1,389**	**425**	**593**	**1,981**	**186**	**1,348**	**330**	**599**	**1,555**
	**I. Communicable, maternal, perinatal, & nutritional**	***5,542***	***2,818***	***2,724***	***225***	***1,265***	***294***	***310***	***874***	***181***	***1,222***	***245***	***396***	***782***
1A01	Tuberculosis	189	118	71	0	3	5	19	91	0	2	4	23	42
1B01	Sexually-transmitted infections	162	78	84	0	10	4	16	48	0	8	3	25	48
1D01	Diarrhoea	506	268	238	2	95	41	31	101	1	90	19	30	99
1E01	Selected vaccine-preventable	52	29	23	0	17	7	3	2	0	16	3	4	0
1F01	Meningitis/encephalitis	122	66	56	0	30	9	12	15	0	25	8	5	18
1G01	Hepatitis	122	87	35	0	6	12	21	48	0	9	6	9	11
1H01	Malaria	1,901	999	902	9	438	140	129	292	1	427	110	110	255
1J01	Respiratory infections	482	254	228	7	108	16	27	103	9	117	16	25	70
1L01	Other infectious and parasitic	1,236	619	617	54	362	57	48	152	51	363	68	54	132
1M01	Maternal	197	0	197	0	0	0	0	0	0	0	1	108	88
1N01	Neonatal	300	167	133	153	167	0	0	0	119	133	0	0	0
	Stillbirth	154	75	79										
1O01	Nutritional	22	11	11	0	7	2	0	2	0	8	1	0	2
1P01	Fever of unknown origin	97	47	50	0	22	1	4	20	0	24	6	3	17
	**II. Non-communicable**	***1,899***	***1,034***	***865***	***2***	***48***	***64***	***121***	***801***	***5***	***62***	***48***	***131***	***624***
	Cancers													
2A01	Oral and upper aerodigestive	19	9	10	0	0	0	0	9	0	0	0	3	7
2A02	Lung and airway	9	9	0	0	0	0	2	7	0	0	0	0	0
2A03	Stomach, liver, and other digestive	97	45	52	0	3	1	3	38	0	2	2	8	40
2A06	Breast	21	1	20	0	0	0	0	1	0	0	0	3	17
2A07	Cervix and uterus	14	0	14	0	0	0	0	0	0	0	0	1	13
2A09	Blood and all others	42	23	19	0	1	3	3	16	0	2	0	3	14
2B01	Diabetes, endocrine, and immune	26	13	13	0	0	0	3	10	0	0	0	4	9
2B02	Sickle-cell anemia	84	41	43	0	7	14	10	10	0	2	15	18	8
	Neuropsychiatric disorders													
2D01	Epilepsy	81	36	45	0	12	9	8	7	0	27	6	5	7
2D02	Other neuropsychiatric	26	12	14	0	1	1	1	9	0	0	0	5	9
2F01	Vision and other sensory loss	34	25	9	0	3	2	4	16	0	2	2	2	3
2F02	Musculoskeletal	6	5	1	0	0	0	1	4	0	0	0	0	1
	Cardiovascular and respiratory diseases													
2G01	Rheumatic heart	4	1	3	0	0	0	1	0	0	0	0	3	0
2G02	Stroke	329	179	150	0	0	1	5	173	0	0	1	3	146
2G03	Ischemic heart	393	205	188	0	0	1	12	192	0	0	3	17	168
2H01	Chronic respiratory	85	39	46	0	2	1	4	32	0	5	4	6	31
	Digestive diseases													
2J01	Gastro-oesophageal	136	76	60	0	0	5	24	47	0	2	3	8	47
2J02	Liver and alcohol-related	153	90	63	0	1	5	11	73	0	1	2	16	44
2J03	Other digestive	200	147	53	0	10	14	24	99	0	7	6	14	26
	Genitourinary diseases													
2K01	Renal failure	70	41	29	0	1	4	3	33	0	0	4	6	19
2K02	Other genitourinary	50	29	21	0	2	1	2	24	0	0	0	6	15
2L01	Congenital anomalies	20	8	12	2	5	2	0	1	5	12	0	0	0
	**III. Injuries**	**731**	**514**	**217**	**2**	**47**	**60**	**142**	**265**	**0**	**37**	**28**	**50**	**102**
3A01	Road traffic accidents	229	160	69	1	7	10	47	96	0	2	7	26	34
3A02	Falls	121	91	30	1	7	18	17	49	0	5	4	5	16
3A03	Drowning	50	37	13	0	6	10	14	7	0	4	7	1	1
3A04	Venomous deaths	84	57	27	0	4	5	15	33	0	2	3	5	17
3A05	Suicide	5	4	1	0	0	1	2	1	0	0	0	1	0
3A06	Interpersonal violence	24	20	4	0	1	1	10	8	0	1	1	0	2
3A07	All other injuries													
	other external causes of accidental injuries	178	119	59	0	21	15	36	47	0	20	6	9	24
	complications of medical and surgical care	40	26	14	0	1	0	1	24	0	3	0	3	8
**4A01**	**IV. Ill-defined or cause unknown**	***202***	***97***	***105***	***0***	**29**	**7**	**20**	**41**	**0**	**27**	**9**	**22**	**47**

Table 3: National Estimated deaths (2020) by age, sex and cause, Sierra Leone

	Sex	Both sexes	Both sexes	Male	Female	Male					Female				
	Age group	Study deaths	Total (all ages)	Total	Total	0-28 days	0-4 years	5-14 years	15-29 years	30-69 years	0-28 days	0-4 years	5-14 years	15-29 years	30-69 years
**Population, thousands (UN 2020)**			7,559	3,776	3,783		519	1,093	1,125	1,039		506	1,045	1,216	1,016
Births (UN 2020)		258,100													
Code	***Cause of death***														
	**All Causes**	**8,374**	**76,939**	**39,062**	**37,877**	**4,250**	**14,665**	**2,611**	**5,514**	**16,272**	**3,768**	**13,058**	**3,616**	**6,740**	**14,464**
	**I. Communicable, maternal, perinatal, & nutritional**	**5,542**	**51,784**	**25,381**	**26,403**	***4,175***	***13,513***	***1,807***	***2,883***	***7,179***	***3,667***	***11,989***	***2,685***	***4,456***	***7,274***
1A01	Tuberculosis	189	1,691	982	709	0	27	31	177	747	0	16	44	259	391
1B01	Sexually-transmitted infections	162	1,482	657	825	0	90	25	149	394	0	64	33	281	446
1D01	Diarrhoea	506	4,440	2,242	2,198	37	872	252	288	830	20	732	208	338	921
1E01	Selected vaccine-preventable	52	446	240	206	0	153	43	28	16	0	128	33	45	0
1F01	Meningitis/encephalitis	122	1,071	559	511	0	269	55	112	123	0	200	88	56	167
1G01	Hepatitis	122	1,058	717	341	0	54	74	195	394	0	72	66	101	102
1H01	Malaria	1901	16,718	8,477	8,241	167	4,019	860	1,200	2,398	20	3,426	1,205	1,238	2,372
1J01	Respiratory infections	482	4,386	2,232	2,153	130	1,037	98	251	846	182	1,046	175	281	651
1L01	Other infectious and parasitic	1236	11,921	5,813	6,108	1,002	3,767	350	446	1,249	1,033	3,527	745	608	1,228
1M01	Maternal	197	2,045	0	2,045	0	0	0	0	0	0	0	11	1,215	819
1N01	Neonatal	300	5,488	2,965	2,523	2,839	2,965	0	0	0	2,411	2,523	0	0	0
	Stillbirth	154	0	0	0										
1O01	Nutritional	22	185	92	94	0	63	12	0	16	0	64	11	0	19
1P01	Fever of unknown origin	97	855	405	449	0	198	6	37	164	0	192	66	34	158
	**II. Non-communicable**	**1899**	**16,909**	**8,548**	**8,361**	***37***	***450***	***393***	***1,125***	***6,579***	***101***	***557***	***526***	***1,474***	***5,804***
	Cancers														
2A01	Oral and upper aerodigestive	19	173	74	99	0	0	0	0	74	0	0	0	34	65
2A02	Lung and airway	9	76	76	0	0	0	0	19	57	0	0	0	0	0
2A03	Stomach, liver, and other digestive	97	877	376	501	0	27	6	28	312	0	16	22	90	372
2A06	Breast	21	200	8	192	0	0	0	0	8	0	0	0	34	158
2A07	Cervix and uterus	14	132	0	132	0	0	0	0	0	0	0	0	11	121
2A09	Blood and all others	42	369	187	182	0	9	18	28	131	0	16	0	34	130
2B01	Diabetes, endocrine, and immune	26	239	110	129	0	0	0	28	82	0	0	0	45	84
2B02	Sickle-cell anemia	84	781	324	457	0	63	86	93	82	0	16	164	203	74
	Neuropsychiatric disorders														
2D01	Epilepsy	81	698	295	403	0	108	55	74	57	0	216	66	56	65
2D02	Other neuropsychiatric	26	238	98	140	0	9	6	9	74	0	0	0	56	84
2F01	Vision and other sensory loss	34	296	208	88	0	27	12	37	131	0	16	22	23	28
2F02	Musculoskeletal	6	51	42	9	0	0	0	9	33	0	0	0	0	9
	Cardiovascular and respiratory diseases														
2G01	Rheumatic heart	4	43	9	34	0	0	0	9	0	0	0	0	34	0
2G02	Stroke	329	2,876	1,474	1,403	0	0	6	46	1,421	0	0	11	34	1,358
2G03	Ischemic heart	393	3,482	1,695	1,787	0	0	6	112	1,577	0	0	33	191	1,563
2H01	Chronic respiratory	85	764	324	440	0	18	6	37	263	0	40	44	68	288
	Digestive diseases														
2J01	Gastro-oesophageal	136	1,216	640	576	0	0	31	223	386	0	16	33	90	437
2J02	Liver and alcohol-related	153	1,361	742	619	0	9	31	102	600	0	8	22	180	409
2J03	Other digestive	200	1,733	1,212	521	0	90	86	223	813	0	56	66	158	242
	Genitourinary diseases														
2K01	Renal failure	70	621	333	288	0	9	25	28	271	0	0	44	68	177
2K02	Other genitourinary	50	447	240	207	0	18	6	19	197	0	0	0	68	140
2L01	Congenital anomalies	20	242	85	157	37	64	12	0	8	101	157	0	0	0
	**III. Injuries**	**731**	**6,421**	**4,307**	**2,114**	***37***	***441***	***369***	***1,320***	***2,177***	***0***	***296***	***307***	***563***	***949***
3A01	Road traffic accidents	229	2,061	1,359	701	19	72	61	437	789	0	16	77	293	316
3A02	Falls	121	1,032	744	289	19	72	111	158	402	0	40	44	56	149
3A03	Drowning	50	432	303	129	0	54	61	130	57	0	32	77	11	9
3A04	Venomous deaths	84	740	477	263	0	36	31	139	271	0	16	33	56	158
3A05	Suicide	5	44	33	11	0	0	6	19	8	0	0	0	11	0
3A06	Interpersonal violence	24	211	174	38	0	9	6	93	66	0	8	11	0	19
3A07	All other injuries														
	other external causes of accidental injuries	178	1,552	1,001	550	0	189	92	335	386	0	160	66	101	223
	complications of medical and surgical care	40	348	215	132	0	9	0	9	197	0	24	0	34	74
**4A01**	**IV. Ill-defined or cause unknown**	**202**	**1,825**	**826**	**999**	***0***	**260**	**43**	**186**	**337**	**0**	**216**	**99**	**248**	**437**

Table 4: Eastern Region burden of disease estimates (2020) for COMSA deaths by age, sex and cause

	Sex	Both sexes	Both sexes	Male	Female	Male					Female				
	Age group	Study deaths	Total (all ages)	Total	Total	0-28 days	0-4 years	5-14 years	15-29 years	30-69 years	0-28 days	0-4 years	5-14 years	15-29 years	30-69 years
**Population, thousands (UN 2020)**			1,752	879	872		112	263	257	247		111	254	281	226
Births (UN 2020)		55,755													
Code	***Cause of death***														
	**All Causes**	**1,169**	**21,413**	**10,920**	**10,493**	**1,000**	**3,458**	**719**	**1,601**	**5,142**	**875**	**3,026**	**965**	**1,915**	**4,588**
	**I. Communicable, maternal, perinatal, & nutritional**	**750**	***13,449***	**6,349**	**7,100**	***941***	***3,048***	***508***	***633***	***2,160***	***875***	***2,796***	***723***	***1,208***	***2,372***
1A01	Tuberculosis	29	546	314	232	0	14	14	0	286	0	13	17	46	157
1B01	Sexually-transmitted infections	31	623	226	397	0	28	0	20	179	0	13	0	205	179
1D01	Diarrhoea	50	863	357	506	0	140	14	59	143	0	140	52	68	246
1E01	Selected vaccine-preventable	7	101	14	86	0	0	14	0	0	0	64	0	23	0
1F01	Meningitis/encephalitis	11	214	107	107	0	0	28	79	0	0	0	17	23	67
1G01	Hepatitis	7	124	107	17	0	14	0	40	54	0	0	17	0	0
1H01	Malaria	211	3,439	1,800	1,639	118	932	240	217	411	0	700	241	228	470
1J01	Respiratory infections	28	479	251	228	0	42	28	20	161	0	76	17	0	134
1L01	Other infectious and parasitic	281	4,997	2,423	2,574	294	1,235	169	198	821	375	1,240	344	228	761
1M01	Maternal	30	678	0	678	0	0	0	0	0	0	0	0	388	291
1N01	Neonatal	23	1,058	558	500	529	558	0	0	0	500	500	0	0	0
	Stillbirth	22	0	0	0										
1O01	Nutritional	3	42	42	0	0	42	0	0	0	0	0	0	0	0
1P01	Fever of unknown origin	17	285	149	135	0	42	0	0	107	0	51	17	0	67
	**II. Non-communicable**	**279**	***5,444***	***2,914***	***2,530***	***59***	***143***	***99***	***494***	***2,178***	***0***	***102***	***138***	***410***	***1,880***
	Cancers														
2A01	Oral and upper aerodigestive	7	139	71	68	0	0	0	0	71	0	0	0	46	22
2A02	Lung and airway	2	38	38	0	0	0	0	20	18	0	0	0	0	0
2A03	Stomach, liver, and other digestive	19	394	125	269	0	0	0	0	125	0	0	0	23	246
2A06	Breast	4	90	0	90	0	0	0	0	0	0	0	0	23	67
2A07	Cervix and uterus	2	45	0	45	0	0	0	0	0	0	0	0	0	45
2A09	Blood and all others	4	82	37	45	0	0	0	20	18	0	0	0	23	22
2B01	Diabetes, endocrine, and immune	5	102	57	45	0	0	0	40	18	0	0	0	0	45
2B02	Sickle-cell anemia	17	320	132	188	0	28	28	40	36	0	0	52	114	22
	Neuropsychiatric disorders														
2D01	Epilepsy	9	138	0	138	0	0	0	0	0	0	76	17	0	45
2D02	Other neuropsychiatric	4	76	54	22	0	0	0	0	54	0	0	0	0	22
2F01	Vision and other sensory loss	2	32	32	0	0	0	14	0	18	0	0	0	0	0
2F02	Musculoskeletal	2	40	18	22	0	0	0	0	18	0	0	0	0	22
	Cardiovascular and respiratory diseases														
2G01	Rheumatic heart	0	0	0	0	0	0	0	0	0	0	0	0	0	0
2G02	Stroke	40	783	430	353	0	0	0	20	411	0	0	17	0	336
2G03	Ischemic heart	59	1,160	650	510	0	0	0	79	571	0	0	17	0	492
2H01	Chronic respiratory	6	123	55	67	0	0	0	20	36	0	0	0	0	67
	Digestive diseases														
2J01	Gastro-oesophageal	18	354	196	157	0	0	14	40	143	0	0	0	23	134
2J02	Liver and alcohol-related	23	469	182	287	0	0	0	40	143	0	0	17	68	201
2J03	Other digestive	38	697	554	143	0	14	42	158	339	0	13	17	68	45
	Genitourinary diseases														
2K01	Renal failure	7	125	125	0	0	0	0	0	125	0	0	0	0	0
2K02	Other genitourinary	8	151	84	68	0	28	0	20	36	0	0	0	23	45
2L01	Congenital anomalies	3	86	73	13	59	73	0	0	0	0	13	0	0	0
	**III. Injuries**	**100**	***1,798***	***1,363***	*436*	***0***	***183***	***85***	***435***	***661***	***0***	***51***	***69***	***137***	***179***
3A01	Road traffic accidents	24	437	316	121	0	14	28	59	214	0	13	17	91	0
3A02	Falls	20	356	237	119	0	28	28	20	161	0	13	17	0	90
3A03	Drowning	10	169	139	30	0	28	14	79	18	0	13	17	0	0
3A04	Venomous deaths	14	265	198	67	0	28	0	99	71	0	0	0	0	67
3A05	Suicide	0	0	0	0	0	0	0	0	0	0	0	0	0	0
3A06	Interpersonal violence	4	71	71	0	0	14	0	40	18	0	0	0	0	0
3A07	All other injuries														
	other external causes of accidental injuries	25	441	366	75	0	70	14	138	143	0	13	17	23	22
	complications of medical and surgical care	3	59	36	23	0	0	0	0	36	0	0	0	23	0
**4A01**	**IV. Ill-defined or cause unknown**	**40**	***722***	***295***	***427***	***0***	**84**	**28**	**40**	**143**	**0**	**76**	**34**	**160**	**157**

Table 5: Northern Region burden of disease estimates (2020) for COMSA deaths by age, sex and cause

	Sex / *Age group*	Both sexes Study deaths	Both sexes Total (all ages)	Male Total	Female Total	Male 0-28 days	0-4 years	5-14 years	15-29 years	30-69 years	Female 0-28 days	0-4 years	5-14 years	15-29 years	30-69 years
**Population, thousands (UN 2020)**			2,668	1,322	1,345		203	420	366	333		195	382	406	361
Births (UN 2020)		97,687													
Code	***Cause of death***														
	**All Causes**	**2,172**	**26,891**	**13,574**	**13,317**	**1,515**	**5,239**	**995**	**2,053**	**5,287**	**1,348**	**4,661**	**1,394**	**2,534**	**4,728**
	**I. Communicable, maternal, perinatal, & nutritional**	***1,480***	***18,441***	***8,722***	***9,719***	***1,489***	***4,827***	***686***	***972***	***2,237***	***1,295***	***4,320***	***1,075***	***1,712***	***2,612***
1A01	Tuberculosis	56	698	414	284	0	12	8	82	312	0	10	0	134	140
1B01	Sexually-transmitted infections	34	444	151	293	0	25	8	41	78	0	10	0	117	166
1D01	Diarrhoea	179	2,082	1,092	989	26	424	181	164	323	26	372	101	134	382
1E01	Selected vaccine-preventable	16	189	91	98	0	62	15	14	0	0	48	17	34	0
1F01	Meningitis/encephalitis	32	367	163	204	0	100	8	0	56	0	115	34	17	38
1G01	Hepatitis	20	228	149	78	0	12	38	55	45	0	19	34	0	25
1H01	Malaria	455	5,384	2,712	2,672	77	1,347	271	369	724	0	1,123	437	436	675
1J01	Respiratory infections	124	1,546	760	786	51	300	60	109	289	106	355	84	117	229
1L01	Other infectious and parasitic	346	4,339	2,052	2,287	308	1,441	98	123	390	211	1,210	353	201	522
1M01	Maternal	61	907	0	907	0	0	0	0	0	0	0	17	520	370
1N01	Neonatal	80	2,017	1,027	990	1,027	1,027	0	0	0	952	990	0	0	0
	Sillbirth	56	0	0	0										
1O01	Nutritional	3	38	25	13	0	25	0	0	0	0	0	0	0	13
1P01	Fever of unknown origin	18	204	86	118	0	50	0	14	22	0	67	0	0	51
	**II. Non-communicable**	**461**	***5,659***	***2,979***	***2,681***	***0***	***212***	***151***	***479***	***2,137***	***53***	***197***	***218***	***571***	***1,695***
	Cancers														
2A01	Oral and upper aerodigestive	5	60	22	38	0	0	0	0	22	0	0	0	0	38
2A02	Lung and airway	2	22	22	0	0	0	0	0	22	0	0	0	0	0
2A03	Stomach, liver, and other digestive	23	282	172	110	0	12	0	14	145	0	0	17	17	76
2A06	Breast	2	25	0	25	0	0	0	0	0	0	0	0	0	25
2A07	Cervix and uterus	3	38	0	38	0	0	0	0	0	0	0	0	0	38
2A09	Blood and all others	4	49	49	0	0	12	0	14	22	0	0	0	0	0
2B01	Diabetes, endocrine, and immune	3	35	22	13	0	0	0	0	22	0	0	0	0	13
2B02	Sickle-cell anemia	12	168	71	97	0	25	8	27	11	0	0	17	67	13
	Neuropsychiatric disorders														
2D01	Epilepsy	20	243	72	170	0	37	8	27	0	0	86	17	67	0
2D02	Other neuropsychiatric	2	25	0	25	0	0	0	0	0	0	0	0	0	25
2F01	Vision and other sensory loss	13	154	98	56	0	12	8	0	78	0	10	34	0	13
2F02	Musculoskeletal	0	0	0	0	0	0	0	0	0	0	0	0	0	0
	Cardiovascular and respiratory diseases														
2G01	Rheumatic heart	0	0	0	0	0	0	0	0	0	0	0	0	0	0
2G02	Stroke	64	768	348	421	0	0	0	14	334	0	0	0	0	421
2G03	Ischemic heart	77	930	497	433	0	0	0	41	456	0	0	0	50	382
2H01	Chronic respiratory	27	336	136	200	0	0	0	14	122	0	10	17	34	140
	Digestive diseases														
2J01	Gastro-oesophageal	56	711	342	369	0	0	30	178	134	0	10	34	84	242
2J02	Liver and alcohol-related	37	447	248	199	0	12	30	27	178	0	0	17	67	115
2J03	Other digestive	77	908	691	217	0	87	60	109	434	0	19	50	84	64
	Genitourinary diseases														
2K01	Renal failure	18	231	100	131	0	12	8	14	67	0	0	17	50	64
2K02	Other genitourinary	13	165	89	76	0	0	0	0	89	0	0	0	50	25
2L01	Congenital anomalies	3	62	0	62	0	0	0	0	0	53	62	0	0	0
	**III. Injuries**	**199**	***2,411***	***1,623***	***788***	***26***	***100***	***143***	***534***	***846***	***0***	***86***	***84***	***235***	***382***
3A01	Road traffic accidents	55	692	430	262	0	0	15	137	278	0	0	34	101	127
3A02	Falls	40	475	371	104	26	63	45	96	167	0	19	17	17	51
3A03	Drowning	6	75	58	17	0	12	8	27	11	0	0	17	0	0
3A04	Venomous deaths	38	457	278	179	0	12	30	68	167	0	10	17	50	102
3A05	Suicide	4	52	35	17	0	0	8	27	0	0	0	0	17	0
3A06	Interpersonal violence	5	59	46	13	0	0	8	27	11	0	0	0	0	13
3A07	All other injuries														
	other external causes of accidental injuries	36	418	302	116	0	12	30	137	122	0	48	0	17	51
	complications of medical and surgical care	15	184	103	81	0	0	0	14	89	0	10	0	34	38
**4A01**	**IV. Ill-defined or cause unknown**	**32**	***379***	***250***	***129***	***0***	**100**	**15**	**68**	**67**	**0**	**58**	**17**	**17**	**38**

Table 6: Western Region burden of disease estimates (2020) for COMSA deaths by age, sex and cause

Code	Cause of death *(Sex / Age group)*	Both sexes Study deaths	Both sexes Total (all ages)	Male Total	Female Total	Male 0-28 days	Male 0-4 years	Male 5-14 years	Male 15-29 years	Male 30-69 years	Female 0-28 days	Female 0-4 years	Female 5-14 years	Female 15-29 years	Female 30-69 years
**Population, thousands (UN 2020)**			1,611	818	793		93	180	291	254		90	193	292	218
Births (UN 2020)		46,831													
Code	***Cause of death***														
	**All Causes**	**755**	**11,893**	**6,185**	**5,707**	**586**	**2,025**	**368**	**926**	**2,866**	**507**	**1,753**	**467**	**1,139**	**2,348**
	**I. Communicable, maternal, perinatal, & nutritional**	**463**	***7,633***	**3,955**	***3,678***	***586***	***1,947***	***261***	***543***	***1,204***	***482***	***1,561***	***360***	***759***	***998***
1A01	Tuberculosis	18	277	153	124	0	0	0	48	105	0	0	0	80	44
1B01	Sexually-transmitted infections	30	443	264	179	0	19	0	48	196	0	33	18	40	88
1D01	Diarrhoea	46	724	363	361	27	104	12	64	183	0	50	0	120	191
1E01	Selected vaccine-preventable	1	17	0	17	0	0	0	0	0	0	17	0	0	0
1F01	Meningitis/encephalitis	11	175	76	99	0	39	24	0	13	0	66	18	0	15
1G01	Hepatitis	8	110	80	29	0	0	12	16	52	0	0	0	0	29
1H01	Malaria	143	2,318	1,206	1,112	0	564	154	240	249	0	349	162	220	382
1J01	Respiratory infections	44	737	435	302	27	221	12	32	170	25	158	0	100	44
1L01	Other infectious and parasitic	88	1,515	878	637	133	580	47	80	170	0	332	162	40	103
1M01	Maternal	11	199	0	199	0	0	0	0	0	0	0	0	140	59
1N01	Neonatal	33	856	399	456	399	399	0	0	0	456	456	0	0	0
	Stillbirth	13	0	0	0										
1O01	Nutritional	2	30	13	17	0	0	0	0	13	0	17	0	0	0
1P01	Fever of unknown origin	15	235	88	147	0	19	0	16	52	0	83	0	20	44
	**II. Non-communicable**	**232**	***3,354***	***1,683***	***1,672***	***0***	***58***	***95***	***208***	***1,322***	***25***	***142***	***72***	***240***	***1,218***
	Cancers														
2A01	Oral and upper aerodigestive	3	42	13	29	0	0	0	0	13	0	0	0	0	29
2A02	Lung and airway	3	42	42	0	0	0	0	16	26	0	0	0	0	0
2A03	Stomach, liver, and other digestive	11	157	79	78	0	0	0	0	79	0	0	0	20	59
2A06	Breast	6	88	0	88	0	0	0	0	0	0	0	0	0	88
2A07	Cervix and uterus	0	0	0	0	0	0	0	0	0	0	0	0	0	0
2A09	Blood and all others	8	123	91	32	0	0	12	16	52	0	17	0	0	15
2B01	Diabetes, endocrine, and immune	2	33	13	20	0	0	0	0	13	0	0	0	20	0
2B02	Sickle-cell anemia	16	256	100	156	0	19	36	32	13	0	0	72	40	44
	Neuropsychiatric disorders														
2D01	Epilepsy	3	51	19	31	0	19	0	0	0	0	17	0	0	15
2D02	Other neuropsychiatric	3	45	25	20	0	0	12	0	13	0	0	0	20	0
2F01	Vision and other sensory loss	3	46	46	0	0	19	0	0	26	0	0	0	0	0
2F02	Musculoskeletal	0	0	0	0	0	0	0	0	0	0	0	0	0	0
	Cardiovascular and respiratory diseases														
2G01	Rheumatic heart	2	36	16	20	0	0	0	16	0	0	0	0	20	0
2G02	Stroke	49	683	340	343	0	0	0	0	340	0	0	0	20	323
2G03	Ischemic heart	53	753	346	407	0	0	0	32	314	0	0	0	40	367
2H01	Chronic respiratory	9	131	39	92	0	0	0	0	39	0	33	0	0	59
	Digestive diseases														
2J01	Gastro-oesophageal	15	224	116	108	0	0	0	64	52	0	0	0	20	88
2J02	Liver and alcohol-related	13	187	121	66	0	0	0	16	105	0	17	0	20	29
2J03	Other digestive	15	213	133	81	0	0	12	16	105	0	17	0	20	44
	Genitourinary diseases														
2K01	Renal failure	6	79	64	15	0	0	12	0	52	0	0	0	0	15
2K02	Other genitourinary	8	109	65	44	0	0	0	0	65	0	0	0	0	44
2L01	Congenital anomalies	4	67	25	42	0	0	12	0	13	25	42	0	0	0
	**III. Injuries**	**43**	***636***	***430***	***206***	***0***	***0***	***12***	***144***	***275***	***0***	***17***	***36***	***80***	***73***
3A01	Road traffic accidents	22	329	198	131	0	0	0	80	118	0	0	18	40	73
3A02	Falls	9	128	108	20	0	0	0	16	92	0	0	0	20	0
3A03	Drowning	3	45	45	0	0	0	0	32	13	0	0	0	0	0
3A04	Venomous deaths	1	13	13	0	0	0	0	0	13	0	0	0	0	0
3A05	Suicide	0	0	0	0	0	0	0	0	0	0	0	0	0	0
3A06	Interpersonal violence	1	16	16	0	0	0	0	16	0	0	0	0	0	0
3A07	All other injuries														
	other external causes of accidental injuries	4	66	12	55	0	0	12	0	0	0	17	18	20	0
	complications of medical and surgical care	3	39	39	0	0	0	0	0	39	0	0	0	0	0
**4A01**	**IV. Ill-defined or cause unknown**	**17**	***269***	***117***	***152***	***0***	**19**	**0**	**32**	**65**	**0**	**33**	**0**	**60**	**59**

Table 7: Southern Region burden of disease estimates (2020) for COMSA deaths by age, sex and cause

	Sex	Both sexes	Both sexes	Male	Female	Male					Female				
	Age group	Study deaths	Total (all ages)	Total	Total	0-28 days	0-4 years	5-14 years	15-29 years	30-69 years	0-28 days	0-4 years	5-14 years	15-29 years	30-69 years
**Population, thousands (UN 2020)**			1,529	757	772		111	229	211	205		109	215	238	211
Births (UN 2020)		57,827													
Code	***Cause of death***														
	**All Causes**	**4,278**	**16,743**	**8,383**	**8,359**	**1,141**	**3,944**	**530**	**934**	**2,976**	**1,047**	**3,619**	**790**	**1,151**	**2,800**
	**I. Communicable, maternal, perinatal, & nutritional**	**2,849**	***11,939***	**5,917**	***6,022***	***1,123***	***3,651***	***364***	***532***	***1,370***	***1,024***	***3,314***	***569***	***761***	***1,378***
1A01	Tuberculosis	86	279	159	120	0	5	8	31	116	0	0	14	34	72
1B01	Sexually-transmitted infections	67	231	106	126	0	23	8	28	48	0	18	10	26	72
1D01	Diarrhoea	231	864	439	425	0	215	38	37	149	0	175	48	49	154
1E01	Selected vaccine-preventable	28	113	77	36	0	55	10	6	6	0	22	10	4	0
1F01	Meningitis/encephalitis	68	261	153	108	0	92	10	25	27	0	40	19	11	38
1G01	Hepatitis	87	290	187	103	0	18	15	43	110	0	31	14	34	24
1H01	Malaria	1092	4,219	2,104	2,115	35	1,134	186	234	551	11	1,057	292	236	530
1J01	Respiratory infections	286	1,165	572	593	35	346	13	49	164	45	350	48	49	147
1L01	Other infectious and parasitic	521	2,464	1,177	1,287	279	860	70	74	173	379	917	86	113	171
1M01	Maternal	95	342	0	342	0	0	0	0	0	0	0	0	199	144
1N01	Neonatal	164	1,465	830	635	775	830	0	0	0	590	635	0	0	0
	Stillbirth	63	0	0	0										
1O01	Nutritional	14	57	17	40	0	9	5	0	3	0	31	5	0	3
1P01	Fever of unknown origin	47	188	97	91	0	64	3	6	24	0	36	24	8	24
	**II. Non-communicable**	**927**	***3,083***	***1,471***	***1,612***	***9***	***100***	***73***	***147***	***1,150***	***22***	***143***	***110***	***251***	***1,108***
	Cancers														
2A01	Oral and upper aerodigestive	4	13	6	7	0	0	0	0	6	0	0	0	4	3
2A02	Lung and airway	2	6	6	0	0	0	0	0	6	0	0	0	0	0
2A03	Stomach, liver, and other digestive	44	153	55	99	0	9	3	6	36	0	9	5	19	65
2A06	Breast	9	31	3	28	0	0	0	0	3	0	0	0	8	21
2A07	Cervix and uterus	9	31	0	31	0	0	0	0	0	0	0	0	4	27
2A09	Blood and all others	26	85	32	54	0	0	5	0	27	0	4	0	8	41
2B01	Diabetes, endocrine, and immune	16	53	21	32	0	0	0	3	18	0	0	0	11	21
2B02	Sickle-cell anemia	39	138	59	79	0	9	20	12	18	0	9	33	26	10
	Neuropsychiatric disorders														
2D01	Epilepsy	49	182	96	86	0	37	20	18	21	0	49	19	4	14
2D02	Other neuropsychiatric	17	58	23	36	0	5	0	3	15	0	0	0	15	21
2F01	Vision and other sensory loss	16	54	35	19	0	5	0	12	18	0	4	0	8	7
2F02	Musculoskeletal	4	12	12	0	0	0	0	3	9	0	0	0	0	0
	Cardiovascular and respiratory diseases														
2G01	Rheumatic heart	2	8	0	8	0	0	0	0	0	0	0	0	8	0
2G02	Stroke	176	559	292	267	0	0	3	9	280	0	0	0	8	260
2G03	Ischemic heart	204	660	295	366	0	0	3	9	283	0	0	10	45	311
2H01	Chronic respiratory	43	148	65	83	0	9	3	6	48	0	9	14	15	44
	Digestive diseases														
2J01	Gastro-oesophageal	47	152	84	68	0	0	0	15	69	0	4	5	4	55
2J02	Liver and alcohol-related	80	255	143	112	0	0	3	18	122	0	0	0	30	82
2J03	Other digestive	70	230	134	96	0	9	5	22	98	0	13	10	19	55
	Genitourinary diseases														
2K01	Renal failure	39	129	59	70	0	0	5	6	48	0	0	14	11	44
2K02	Other genitourinary	21	67	32	35	0	0	3	3	27	0	0	0	8	27
2L01	Congenital anomalies	10	61	20	40	9	18	3	0	0	22	40	0	0	0
	**III. Injuries**	**389**	***1,308***	***825***	***484***	***9***	***128***	***85***	***221***	***390***	***0***	***103***	***81***	***98***	***202***
3A01	Road traffic accidents	128	421	285	136	9	32	15	89	149	0	4	14	53	65
3A02	Falls	52	165	108	57	0	5	25	25	54	0	9	10	11	27
3A03	Drowning	31	109	64	45	0	14	20	18	12	0	13	24	4	3
3A04	Venomous deaths	31	103	61	42	0	5	3	15	39	0	4	10	8	21
3A05	Suicide	1	3	3	0	0	0	0	0	3	0	0	0	0	0
3A06	Interpersonal violence	14	46	33	13	0	0	0	15	18	0	4	5	0	3
3A07	All other injuries														
	other external causes of accidental injuries	113	398	233	165	0	69	23	58	83	0	58	19	23	65
	complications of medical and surgical care	19	63	37	26	0	5	0	0	33	0	9	0	0	17
**4A01**	**IV. Ill-defined or cause unknown**	**113**	***412***	***171***	***241***	***0***	**64**	**8**	**34**	**66**	**0**	**58**	**29**	**41**	**113**
